# Genetic, metabolomic and transcriptomic analyses of the de novo L-cysteine biosynthetic pathway in the enteric protozoan parasite *Entamoeba histolytica*

**DOI:** 10.1038/s41598-017-15923-3

**Published:** 2017-11-15

**Authors:** Ghulam Jeelani, Dan Sato, Tomoyoshi Soga, Tomoyoshi Nozaki

**Affiliations:** 1 Department of Biomedical Chemistry, Graduate School of Medicine, The University of Tokyo, 7-3-1 Hongo, Bunkyo-ku, Tokyo, 113-0033 Japan; 2 Department of Parasitology, National Institute of Infectious Diseases, 1-23-1 Toyama, Shinjuku, Tokyo, 162-8640 Japan; 3 Institute for Advanced Biosciences, Keio University, Tsuruoka, Yamagata, Japan; 4 Graduate School of Life and Environmental Sciences, University of Tsukuba, 1-1-1 Tennodai, Tsukuba, Ibaraki, 305-8572 Japan; 5 Present Address: Graduate School of Science and Technology, Department of Applied Biology, Kyoto Institute of Technology, Kyoto, 606-8585 Japan

## Abstract

The de novo L-cysteine biosynthetic pathway is critical for the growth, antioxidative stress defenses, and pathogenesis of bacterial and protozoan pathogens, such as *Salmonella typhimurium* and *Entamoeba histolytica*. This pathway involves two key enzymes, serine acetyltransferase (SAT) and cysteine synthase (CS), which are absent in mammals and therefore represent rational drug targets. The human parasite *E*. *histolytica* possesses three SAT and CS isozymes; however, the specific roles of individual isoforms and significance of such apparent redundancy remains unclear. In the present study, we generated *E*. *histolytica* cell lines in which CS and SAT expression was knocked down by transcriptional gene silencing. The strain in which *CS1*, 2 and 3 were simultaneously silenced and the *SAT*3 gene-silenced strain showed impaired growth when cultured in a cysteine lacking BI-S-33 medium, whereas silencing of *SAT1* and *SAT2* had no effects on growth. Combined transcriptomic and metabolomic analyses revealed that, CS and SAT3 are involved in S-methylcysteine/cysteine synthesis. Furthermore, silencing of the *CS1-3* or *SAT3* caused upregulation of various iron-sulfur flavoprotein genes. Taken together, these results provide the first direct evidence of the biological importance of SAT3 and CS isoforms in *E*. *histolytica* and justify the exploitation of these enzymes as potential drug targets.

## Introduction

Critical metabolic pathways that are unique to pathogens and are significantly divergent from their hosts are rational targets for the development of new chemotherapeutic agents. In particular, sulfur-containing amino acid metabolism, particularly the de novo L-cysteine biosynthetic pathway, is a promising target for drug development against bacterial and parasitic infections, such as those caused by *Mycobacterium tuberculosis*, *Salmonella typhimurium*, and *Entamoeba histolytica* ^1–6^.

Amebiasis is an intestinal infection caused by the protozoan pathogen *E*. *histolytica* and is widespread worldwide [CDC, https://www.cdc.gov/parasites/amebiasis/index.html], particularly in countries with inadequate sewage treatment and poor water quality^7^. According to the WHO, an estimated 50 million people are infected with *E*. *histolytica* worldwide, resulting in 40,000–100,000 deaths annually^8^. Metronidazole is the drug of choice for treating amebiasis despite its low efficacy against asymptomatic cyst carriers^1^. Moreover, metronidazole is also teratogenic and causes adverse side effects, such as nausea, vomiting, headache, insomnia, dizziness, drowsiness and hypersensitivity reactions (urticaria, pruritus, erythematous rash)^9^. In addition, *E*. *histolytica* is capable of tolerating sub-therapeutic levels of metronidazole *in vitro* ^10,11^. Therefore, new drugs that target parasite-specific metabolic pathways and enzymes distinct from those targeted by metronidazole are urgently needed.

Sulfur-containing amino acid metabolism in *E*. *histolytica* differs markedly from that in humans with respect to three main features: (i) the absence of forward and reverse transsulfuration pathways and thus does not convert L-methionine to L-cysteine^12,13^ or vice versa; (ii) the presence of a sulfur-assimilatory de novo L-cysteine biosynthetic pathway^14–16^; and (iii) the presence of a unique enzyme, methionine γ-lyase (MGL), which is involved in the degradation of sulfur-containing amino acids^17–19^. As MGL and two enzymes involved in the cysteine biosynthetic pathway, serine *O*-acetyltransferase (SAT) and cysteine synthase (CS, *O*-acetylserine sulfhydrylase), are absent in mammals, these enzymes are potential suitable targets for chemotherapeutic agents against amebiasis.

**Figure 1:**
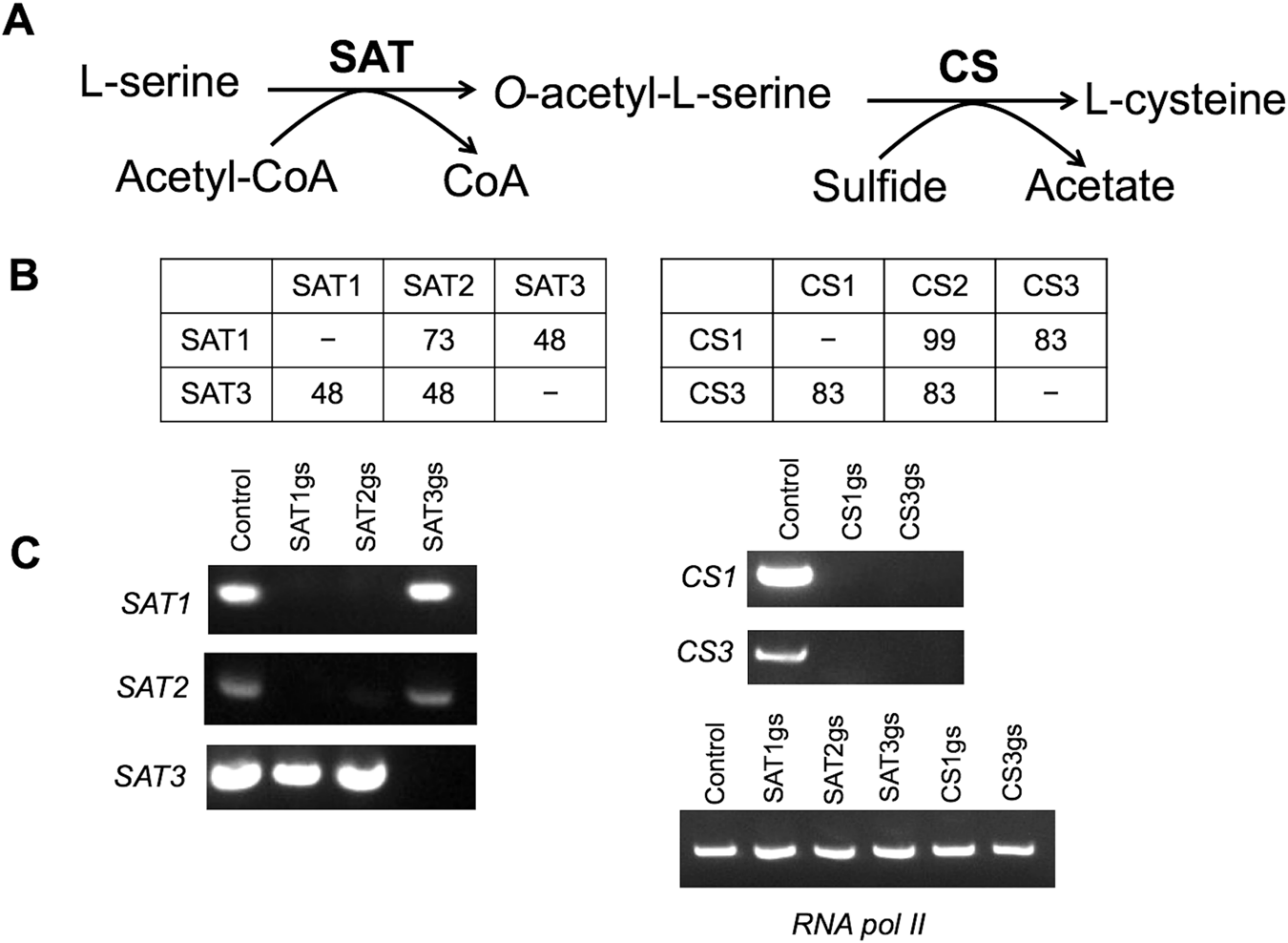
Epigenetic repression of cysteine biosynthesis pathway genes in *E*. *histolytica* G3 strain. (**A**) Scheme of the sulfur assimilatory de novo cysteine biosynthetic pathway in *E*. *histolytica*. Abbreviations: SAT, serine O-acetyltransferase (EC 2.3.1.30); CS, cysteine synthase (O-acetyl-L-serine sulfhydrylase, EC 2.5.1.47); and CoA, coenzyme A. (**B**) Percent amino acid identity among *E*. *histolytica* SAT and CS isoforms by ClustalW multiple sequence alignment score. GenBank accession numbers: SAT1, BAA82868; SAT2, XP_650001; SAT3, XP_656373; CS1, XP_650965; CS2, XP_648291; and CS3, XP_653246. (**C**) Semi-quantitative RT-PCR analysis of *SAT1*, *SAT2*, *SAT3*, *CS1* and *CS3* gene transcript levels in trophozoites of strain G3 transfected with either empty vector (psAP2G) or the constructed gene silencing plasmids (psAP2G-SAT1, psAP2G-SAT2, psAP2G-SAT3, psAP2G-CS1 and psAP2G-CS3). cDNA from the generated cell lines (psAP2G, SAT1gs, SAT2gs, SAT3gs, CS1gs and CS3gs) was subjected to 30 cycles of PCR using specific primers for the *SAT1*, *SAT2*, *SAT3*, *CS1* and *CS3* genes. RNA polymerase II served as a control. PCR analysis of samples without reverse transcription was used to exclude the possibility of genomic DNA contamination.

The cysteine biosynthetic pathway plays an important role in the incorporation of inorganic sulfur into organic compounds^1^ and has been extensively studied in bacteria, plants, and protozoa^20–24^. In this pathway, SAT (EC 2.3.1.30) catalyzes the formation of *O*-acetyl-L-serine (OAS) from L-serine and acetyl-CoA^15,16^ (Fig. 1A). CS [*O*-acetyl-L-serine (thiol)-lyase] (EC 4.2.99.8) then catalyzes the production of L-cysteine/S-methylcysteine (SMC) through the modification of sulfide/methanthiol with the alanyl moiety of *O*-acetylserine^13,14^. However, using a metabolomics approach, we previously showed that CS enzymes in *E*. *histolytica* trophozoites cultured in the absence of exogenous L-cysteine are predominantly involved in SMC formation, but not L-cysteine^13^. *E*. *histolytica* SAT and CS have several unique features with respect to localization, complex formation and homology. For example, isozymes of SAT (EhSAT1-3) and CS (EhCS1-3) are localized to the cytosol^14–16^, whereas plant isoforms of SAT and CS are found in the mitochondria, plastids, and cytosol^25^. In addition, EhCS1 and EhSAT1 do not form a heteromeric complex^26^, whereas bacterial and plant SAT and CS form complexes that are involved in cross-talk between sulfur assimilation, carbon and nitrogen metabolism via the generation of OAS^27^. Further, EhSAT1-3 are biochemically divergent, showing 48–73% mutual sequence identity (Fig. 1B) and markedly different sensitivities to allosteric feedback by L-cysteine^16^. EhCS1-3 also exhibit sequence divergence with CS1 and CS2 being identical with the exception of two amino acid changes and CS3 having 83% amino acid identity with CS1 and CS2 (Fig. 1B).

Although the sulfur-assimilatory cysteine biosynthetic pathway in plants, bacteria, and protozoa has been extensively studied and exploited for drug development, the role of individual SAT and CS isozymes and significance of the apparent redundancy of this pathway in *E*. *histolytica* remain to be elucidated. In the present study, we investigated the role of the cysteine biosynthesis pathway in *E*. *histolytica* using parasites in which genes for the enzymes involved in cysteine biosynthesis were silenced by antisense RNA-mediated transcriptional attenuation. Using transcriptomic and metabolomic analyses, we demonstrated that EhCS and EhSAT3 are critical for SMC/cysteine production and cell growth. Furthermore, we examined the fate of SMC unique metabolite in *E*. *histolytica* and revealed that this unique metabolite is involved in the antioxidative stress mechanism.

## Results

### Establishment of *CS* and *SAT* gene-silenced strains

To investigate the role of the L-cysteine biosynthesis pathway in *E*. *histolytica*, we utilized antisense small RNA-mediated epigenetic gene silencing to repress the *CS1/2* (CS1 and CS2 are 99% identical at the amino acid level), *CS3*, *SAT1*, *SAT2*, and *SAT3* genes in *E*. *histolytica* strain G3 (Fig. 1C)^28,29^. In the *CS1/2* and *CS3* gene-silenced strains, *CS1/2* and *CS3* gene expression were simultaneously repressed, likely due to the high sequence similarity (83% amino acid identity) between these genes (Fig. 1B). Similarly, the *SAT1* and *SAT2* genes, whose products share 73% amino acid identity, were simultaneously silenced in the *SAT1* and *SAT2* gene-silenced transformants (Fig. 1B), whereas the *SAT3* gene was not silenced in either of these transformants because of low (48% at the amino acid level) identity between SAT1 and SAT3 and between SAT2 and SAT3. In the *SAT3* gene-silenced strain, only the *SAT3* gene was silenced (Fig. 1C), and neither *SAT1* nor *SAT2* was affected. In subsequent analyses, the *SAT1/2* and *CS1/3* gene-silenced transformants, designated SAT1/2gs and CSgs, respectively, were used for further analyses.

### Effects of *CS* and *SAT* gene silencing on *E*. *histolytica* growth

**Figure 2:**
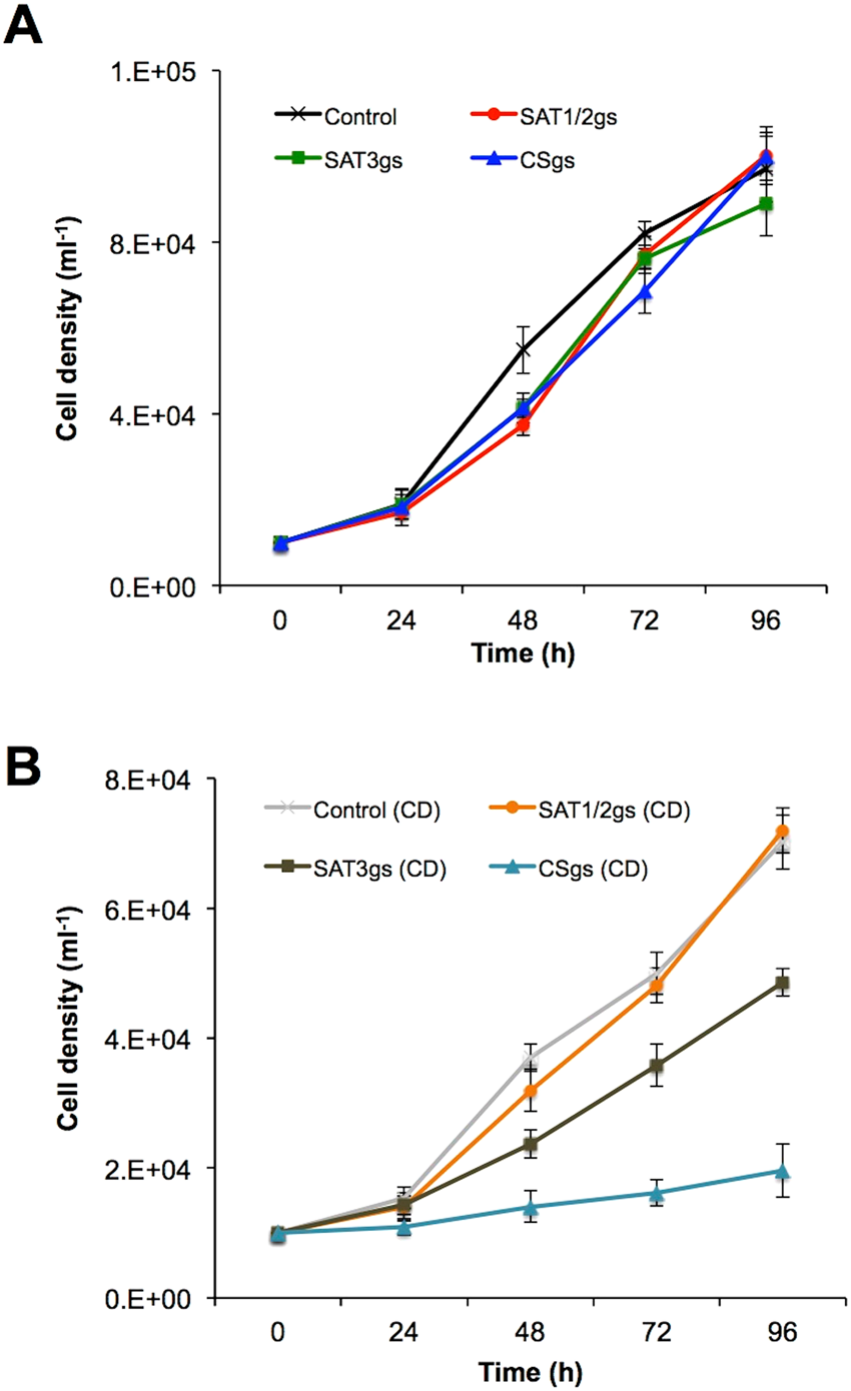
Effect of *SAT1/2*, *SAT3* and *CS* gene silencing on the growth of trophozoites cultured under normal (**A**) and L-cysteine lacking (CD) BI-S-33 medium (**B**). Approximately 6000 amoebae in the logarithmic growth phase were inoculated into 6 mL fresh culture medium and amoebae were then counted every 24 h. Data shown are the means ± standard deviations of five biological replicates.

To examine if the L-cysteine biosynthesis plays a role in the proliferation of *E*. *histolytica*, the growth kinetics of trophozoites of the gene-silenced and control transformants (cell line transfected with psAP2G plasmid) were compared in normal BI-S-33 medium containing 8 mM L-cysteine (Fig. 2A) or BI-S-33 medium without L-cysteine which we called as L-cysteine lacking medium (Fig. 2B). However this medium may still contain trace amounts of cysteine from yeast extract and/or tryptone. When cultured in L-cysteine lacking medium, *CS* gene-disrupted transformants displayed a severe growth defect, whereas *SAT3* gene-disrupted transformants showed a mild growth defect (Fig. 2B). In contrast, SAT1/2gs transformants appeared to grow normally in L-cysteine lacking medium (Fig. 2B). However, in normal BI-S-33 medium, none of the gene-silenced strains showed defective growth (Fig. 2A). These results indicate that CS and SAT3 are essential for growth in the absence of exogenous L-cysteine and therefore contribute to cell proliferation.

### Metabolomic analysis of gene-silenced transformants cultured in normal and L-cysteine lacking BI-S-33 medium

**Figure 3:**
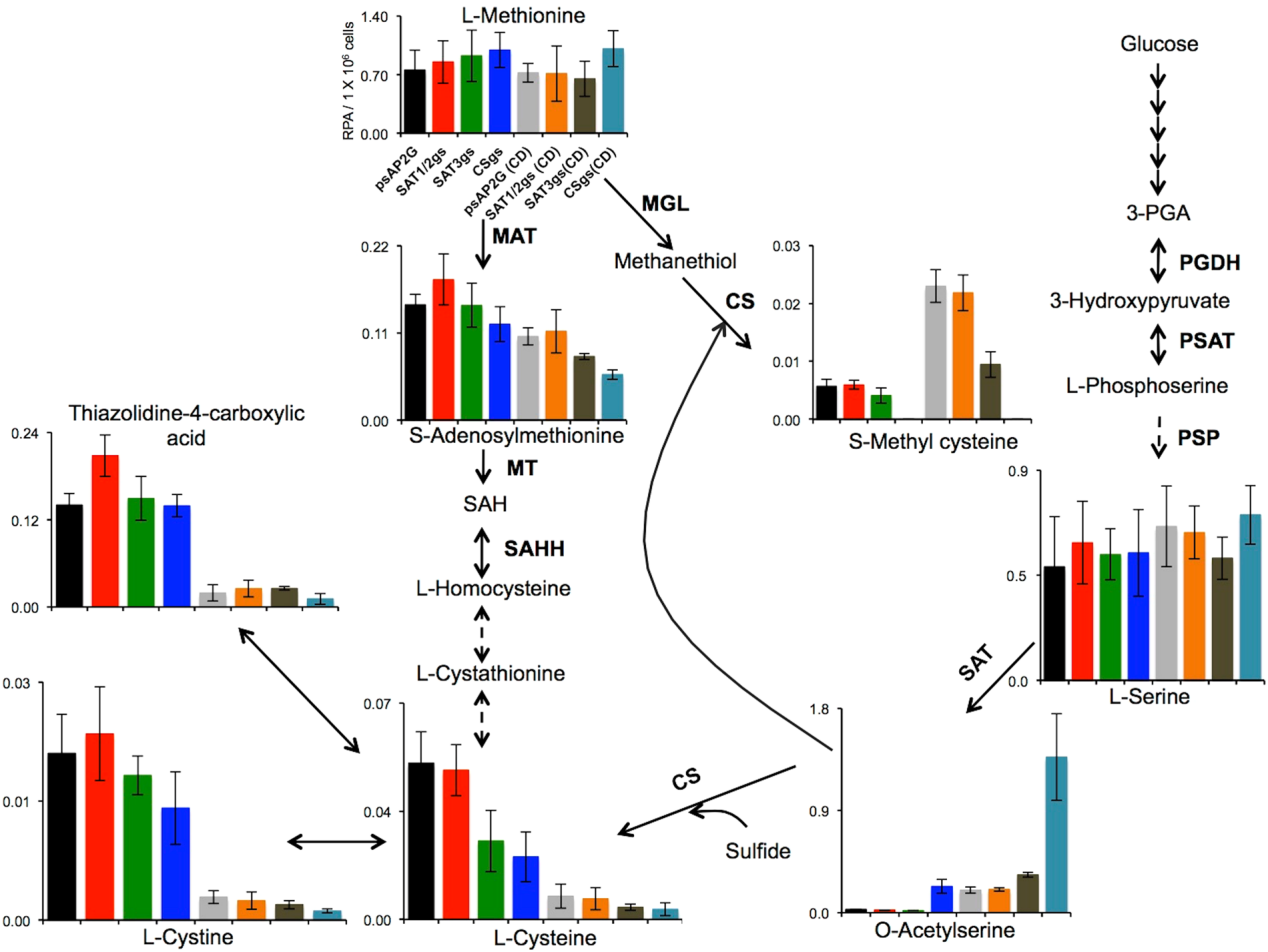
Effect of *SAT1/2*, *SAT3* and *CS3* gene silencing and L-cysteine depletion on sulfur-containing amino acid metabolism. Levels of metabolites extracted from *SAT1/2*, *SAT3* and *CS* gene silenced (SAT1/2gs, SAT3gs, and CSgs) and control (harboring plasmid psAP2G) strains cultured under normal and L-cysteine lacking (CD) BI-S-33 medium conditions is shown. Data shown are the means ± standard deviations of three biological replicates. In the metabolic pathway schemes, solid arrows represent the steps catalyzed by the enzymes whose encoding genes are present in the *E*. *histolytica* genome, whereas an arrow with a dashed line indicates those likely absent in the genome or not yet identified so far. Abbreviations: RPA, relative peak area; 3PGA, 3-phosphoglycerate; SAH, *S*-adenosylhomocysteine; PGDH, phosphoglycerate dehydrogenase (EC 1.1.1.95); PSAT, phosphoserine aminotransferase (EC 2.6.1.52); PSP, phosphoserine phosphatase (EC 3.1.3.3); MGL, methionine γ-lyase (L-methioninase, EC 4.4.1.11); SAT, serine O-acetyltransferase (EC 2.3.1.30); CS, cysteine synthase (O-acetyl-L-serine sulfhydrylase, EC 2.5.1.47); MAT, methionine adenosyltransferase (S-adenosyl-L-methionine synthetase, EC 2.5.1.6); MT, various methyltransferases (EC 2.1.1.X); and SAHH, adenosylhomocysteinase (S-adenosyl-L-homocysteine hydrolase, EC 3.3.1.1).

A total of 48 intermediary metabolites, including amino acids, nucleotides, and organic acids, were measured by CE-TOFMS-based metabolomic analysis in the SAT1/2gs, SAT3gs and CSgs transformants under different culture conditions (Supplementary Table S2). Silencing of the *CS* genes caused drastic changes in the metabolites involved in sulfur-containing amino acid metabolism (Fig. 3). Specifically, the L-cysteine concentration in CSgs trophozoites was approximately 60% lower than that in control trophozoites when cultured under normal BI-S-33 containing 8 mM L-cysteine and L-cysteine-lacking BI-S-33 medium, consistent with the speculation that CS is involved in L-cysteine production. *CS* gene silencing also resulted in a marked increase in OAS, an activated form of L-serine that is synthesized from L-serine and acetyl-CoA by SAT, in both normal and L-cysteine lacking conditions. In addition, SMC formation was completely abolished by CS gene silencing (Fig. 3), suggesting that CS enzymes are indispensable for SMC production.

In contrast to *CS*, silencing of *SAT1/2* did not markedly alter the levels of sulfur-containing metabolites, particularly OAS, SMC, L-cysteine and L-methionine, in *E*. *histolytica*, suggesting that SAT3 can compensate for the loss of SAT1/2. However, upon silencing of the *SAT3* gene, the levels of SMC and L-cysteine were decreased approximately 40–50% compared to the control strain despite the presence of high levels of the precursor metabolite OAS, which is formed by SAT1 and SAT2 in strain SAT3gs. The reduced level of SMC/cysteine with the concurrent higher OAS level (approximately 60% increase) in strain SAT3gs may be due to a decreased level of CS protein in strain SAT3gs. To investigate this possibility, we examined CS expression at the protein level in the SAT1/2gs, SAT3gs, and control transformant strains. Immunoblot analysis using anti-rEhCS1, anti-rEhCS3, and anti-rEhCPBF1^30^ antibodies showed that the relative amounts of these proteins were comparable between these strains (Supplementary Fig. S1), suggesting that SAT3 may positively regulate CS activity, but not gene expression or protein stability, whereas SAT1/2 do not regulate CS activity.

### Gene silencing of *SAT1/2*, *SAT3*, or *CS1-3* caused global transcriptomic changes

To determine if the silencing of the *CS* and *SAT* genes affected the expression of other genes, global gene expression in the SAT1/2gs SAT3gs and CSgs transformants was analyzed using a whole-genome DNA microarray. However, the analysis revealed that after the removal of redundant or obsolete genes (those represented with probe sets with ‘_x_at’ and those for which corresponding NCBI entries were removed after genome reannotation)^31^, only a limited number of genes had three-fold or higher changes in expression (Supplementary Table S3).

In CSgs, 34 genes were up-regulated and 25 genes were down-regulated when compared to the control (Table 1). *CS1*-*3* transcript levels were reduced by 104, 128 and 20.4 fold, respectively, in CSgs. Among the genes that were significantly down-regulated included those encoding for several hypothetical proteins (EHI_020830, EHI_196760, and EHI_066720), Rab family GTPase, RabH2^32^ (EHI_128180), and a nonpathogenic pore-forming peptide precursor (EHI_169350), which may belong in the saposin-like protein^31^ (SAPLIP1) family. In contrast, Ras family GTPase (EHI_074750_at), methylene-fatty-acyl-phospholipid synthase (EHI_153710_at), and deoxyuridine 5′ triphosphate nucleotide hydrolase domain-containing protein (EHI_091670_at) were up-regulated in all three gene-silenced transformants (Supplementary Table S3), suggesting that the increased expression of these genes may compensate for the impairment of the cysteine biosynthetic pathway. The most highly upregulated gene related to sulfur metabolism in CSgs was a gene encoding a member of the NADPH-dependent FMN reductase domain-containing protein family (Table 1). Genes encoding NADPH-dependent oxidoreductase 2 (EHI_045340), which was previously shown to be involved in cystine reduction^33^, was also upregulated in CSgs strain (Table 1).

**Table 1:** List of genes down and up regulated ≥3 fold upon CS gene silencing.

ProbeSetID	Accession Numbers	Common Name	Basal Expression (log_2_)	Fold change	Regulation	p-value
EHI_160930_s_at	XM_643199	cysteine synthase 2	11.4	127.7	down	0.000
EHI_024230_s_at	XM_645873	cysteine synthase 1	10.4	104.3	down	0.019
EHI_060340_at	XM_648154	cysteine synthase 3	6.7	20.4	down	0.000
EHI_020830_s_at	XM_001913952	hypothetical protein	7.8	19.9	down	0.016
EHI_128180_s_at	XM_649666	Rab family GTPase	8.1	14.9	down	0.004
EHI_196760_s_at	XM_643708	hypothetical protein	7.7	10.2	down	0.005
EHI_133210_s_at	XM_001914244	peptidase S54 (rhomboid) family protein	7.9	6.1	down	0.009
EHI_169350_at	XM_650744	nonpathogenic pore-forming peptide precursor, putative	7.6	6.0	down	0.005
EHI_056700_at	XM_643998	hypothetical protein	7.1	5.1	down	0.006
EHI_066720_at	XM_646043	hypothetical protein, conserved	7.2	5.0	down	0.000
EHI_161970_at	XM_644065	leucyl-tRNA synthetase, putative	8.7	4.6	down	0.009
EHI_153670_at	XM_651265	U1 small nuclear ribonucleoprotein subunit, putative	7.9	4.0	down	0.004
EHI_059870_s_at	XM_647804	WH2 domain containing protein	9.8	4.0	down	0.000
EHI_187280_at	XM_651366	transcription initiation factor SPT5, putative	8.7	3.8	down	0.015
EHI_185620_at	XM_644513	protein kinase, putative	5.7	3.8	down	0.033
EHI_029600_at	XM_644990	leucine rich repeat-containing protein	7.9	3.7	down	0.002
EHI_197440_at	XM_646593	hypothetical protein	10.6	3.6	down	0.000
EHI_180940_at	XM_646942	lipase, putative	4.9	3.5	down	0.010
EHI_060350_at	XM_648153	splicing factor Prp8, putative	6.9	3.5	down	0.016
EHI_155220_at	XM_643278	T-complex protein 1, alpha subunit, putative	8.3	3.4	down	0.010
EHI_065670_at	XM_648551	cation-transporting P-typeATPase, putative	10.8	3.3	down	0.019
EHI_178610_at	XM_651172	tyrosine kinase, putative	7.6	3.3	down	0.047
EHI_177660_at	XM_650844	isoleucyl-tRNA synthetase, putative	8.6	3.2	down	0.006
EHI_005050_at	XM_647746	sucrose transporter, putative	4.5	3.2	down	0.039
EHI_167130_at	XM_649685	filopodin, putative	9.3	3.2	down	0.000
EHI_074750_at	XM_644490	Ras family GTPase	5.5	10.1	up	0.003
EHI_091670_at	XM_644055	deoxyuridine 5′triphosphate nucleotidohydrolase domain containing protein	2.3	4.9	up	0.009
EHI_126550_at	XM_643463	AIG1 family protein, putative	6.5	4.6	up	0.003
EHI_022270_s_at	XM_644774	NADPH-dependent FMN reductase domain containing protein	7.2	4.5	up	0.001
EHI_159660_at	XM_645152	hypothetical protein	5.9	4.4	up	0.005
EHI_151780_at	XM_652309	hypothetical protein	2.4	4.1	up	0.017
EHI_067720_s_at	XM_643101	NADPH-dependent FMN reductase domain containing protein	7.3	4.0	up	0.015
EHI_045340_s_at	XM_648481	NADPH-dependent oxidoreductases 2	9.0	4.0	up	0.003
EHI_022600_s_at	XM_643169	NADPH-dependent FMN reductase domain containing protein	7.0	4.0	up	0.003
EHI_134710_at	XM_647029	hypothetical protein	4.4	4.0	up	0.013
EHI_059320_s_at	XM_001914076	hypothetical protein	2.6	3.9	up	0.024
EHI_072960_s_at	XM_001914509	deoxyuridine 5′triphosphate nucleotidohydrolase domain containing protein	4.3	3.9	up	0.010
EHI_153710_at	XM_001913338	methylene-fatty-acyl-phospholipid synthase, putative	6.5	3.9	up	0.007
EHI_182540_at	XM_651612	Protein tyrosine phosphatases domain containing protein	3.8	3.9	up	0.000
EHI_150660_s_at	XM_642980	hypothetical protein	3.7	3.9	up	0.028
EHI_022990_at	XM_648401	hypothetical protein	4.8	3.8	up	0.008
EHI_174570_at	XM_648228	hypothetical protein	2.3	3.7	up	0.001
EHI_025710_at	XM_644279	iron-sulfur flavoprotein, putative	5.5	3.7	up	0.001
EHI_146130_at	XM_644793	hypothetical protein	3.3	3.7	up	0.035
EHI_046630_at	XM_645444	Rho family GTPase	4.1	3.7	up	0.011
EHI_172510_at	XM_643770	acid sphingomyelinase-like phosphodiesterase 3a precursor, putative	3.5	3.6	up	0.000
EHI_174970_at	XM_648244	hypothetical protein	5.4	3.5	up	0.010
EHI_103260_s_at	XM_001913434	NADPH-dependent FMN reductase domain containing protein	7.3	3.5	up	0.007
EHI_181710_s_at	XM_001914510	NADPH-dependent FMN reductase domain containing protein	7.3	3.5	up	0.013
EHI_121870_at	XM_646700	ADP-ribosylation factor 1, putative	5.4	3.5	up	0.000
EHI_069590_at	XM_001913469	hypothetical protein	5.1	3.4	up	0.005
EHI_125910_at	XM_651393	double-strand break repair protein MRE11, putative	3.1	3.4	up	0.006
EHI_052130_at	XM_650257	PQ loop repeat protein	4.6	3.3	up	0.018
EHI_155430_s_at	XM_650657	hypothetical protein	4.7	3.3	up	0.012
EHI_001800_at	XM_644530	hypothetical protein	4.7	3.3	up	0.019
EHI_192550_at	XM_001913649	hypothetical protein	2.3	3.2	up	0.009
EHI_101260_at	XM_651922	Ras family GTPase	5.6	3.1	up	0.008
EHI_105080_at	XM_648821	zinc finger protein, putative	8.2	3.1	up	0.031
EHI_159470_at	XM_648174	hypothetical protein	2.7	3.0	up	0.018

In strain SAT1/2gs, 39 genes were up-regulated and 13 genes were down-regulated compared to the control strain (Table 2). The *SAT1* and *SAT2* transcript levels were reduced by 529 and 4.2 fold, respectively, whereas the expression of the *SAT3* gene remained unchanged. The genes encoding phosphoserine aminotransferase (EHI_026360), which catalyzes the formation of L-phosphoserine from 3-phosphohydroxypyruvate in the phosphorylated pathway of L-serine biosynthesis^34^, were down-regulated more than five fold (Table 2). Among the most highly upregulated genes was sulfotransferase (EHI_031640), which was up-regulated more than 8 fold, and Fe hydrogenase, which was induced more than 4 fold in strain SAT1/2gs (Table 2). In strain SAT3gs, 16 genes were up-regulated and 19 were down-regulated compared to the control (Table 3). The most highly repressed gene in SAT3gs was SAT3, which had 187-fold lower transcript levels compared the control, whereas *SAT1* and *SAT2* gene expression remained unchanged. Among the genes that were up-regulated by *SAT3* gene silencing were several genes encoding NADPH-dependent FMN reductase domain-containing protein and iron-sulfur flavoprotein (ISF) genes, which were among the most highly up-regulated genes by *CS3* gene silencing (Table 1).

**Table 2:** List of genes down and up regulated ≥3 fold upon SAT1/2 gene silencing.

ProbeSetID	Accession Number	Common Name	Basal Expression (log_2_)	Fold change	Regulation	p-value
EHI_202040_at	AB023954	serine acetyltransferase 1	12.2	528.6	down	0.002
EHI_020830_s_at	XM_001913952	hypothetical protein	7.8	11.7	down	0.001
EHI_196760_s_at	XM_643708	hypothetical protein	7.7	10.6	down	0.014
EHI_026360_s_at	XM_650291	phosphoserine aminotransferase, putative	8.5	5.1	down	0.004
EHI_187090_at	XM_651385	Rab family GTPase	10.8	4.4	down	0.007
EHI_021570_at	XM_644909	serine acetyltransferase 2	4.2	3.6	down	0.005
EHI_169350_at	XM_650744	nonpathogenic pore-forming peptide precursor, putative	7.6	3.5	down	0.024
EHI_066720_at	XM_646043	hypothetical protein, conserved	7.2	3.3	down	0.000
EHI_003950_at	XM_643818	hypothetical protein	8.3	3.2	down	0.027
EHI_094060_s_at	XM_001913553	actin binding protein, putative	10.1	3.2	down	0.015
EHI_199170_s_at	XM_648207	hypothetical protein, conserved	6.4	3.1	down	0.049
EHI_183120_s_at	XM_649872	centromeric protein E, putative	8.0	3.1	down	0.014
EHI_073980_s_at	XM_648468	surface antigen ariel1, putative	4.0	3.0	down	0.001
EHI_031640_at	XM_648447	sulfotransferase, putative	7.5	8.5	up	0.000
EHI_193640_s_at	XM_643661	hypothetical protein	2.3	8.2	up	0.002
EHI_074750_at	XM_644490	Ras family GTPase	5.5	7.8	up	0.004
EHI_018140_s_at	XM_001914260	deoxyuridine 5′-triphosphate nucleotidohydrolase domain containing protein	5.5	6.7	up	0.002
EHI_072960_s_at	XM_001914509	deoxyuridine 5′triphosphate nucleotidohydrolase domain containing protein	4.3	6.0	up	0.007
EHI_070690_at	XM_001913839	Ras GTPase domain conting protein	2.3	5.7	up	0.020
EHI_046630_at	XM_645444	Rho family GTPase	4.1	5.3	up	0.005
EHI_174970_at	XM_648244	hypothetical protein	5.4	5.2	up	0.000
EHI_126550_at	XM_643463	AIG1 family protein, putative	6.5	5.1	up	0.002
EHI_068270_s_at	XM_646627	Rho guanine nucleotide exchange factor, putative	4.1	4.9	up	0.001
EHI_146680_s_at	XM_001914548	hypothetical protein	2.4	4.8	up	0.009
EHI_004520_at	XM_651631	hypothetical protein	5.1	4.6	up	0.013
EHI_046040_s_at	XM_645992	hypothetical protein	5.0	4.5	up	0.001
EHI_095910_at	XM_001913730	lipase, putative	4.7	4.4	up	0.001
EHI_134850_at	XM_647045	Fe-hydrogenase, putative	7.5	4.4	up	0.011
EHI_120580_at	XM_646886	hypothetical protein	3.3	4.3	up	0.026
EHI_067910_at	XM_651687	competence protein ComEC, putative	6.3	4.3	up	0.011
EHI_146130_at	XM_644793	hypothetical protein	3.3	4.1	up	0.001
EHI_151440_at	XM_652272	cysteine proteinase, putative	7.5	4.1	up	0.000
EHI_182540_at	XM_651612	Protein tyrosine phosphatases domain containing protein	3.8	4.1	up	0.001
EHI_074580_at	XM_645859	hypothetical protein	3.2	3.9	up	0.025
EHI_019630_at	XM_643344	hypothetical protein	6.9	3.9	up	0.002
EHI_123700_at	XM_648695	hypothetical protein	3.7	3.9	up	0.019
EHI_028940_at	XM_645826	hypothetical protein	8.8	3.8	up	0.000
EHI_191730_at	XM_643923	cysteine protease binding protein family 10	5.3	3.7	up	0.017
EHI_022990_at	XM_648401	hypothetical protein	4.8	3.7	up	0.002
EHI_153710_at	XM_001913338	methylene-fatty-acyl-phospholipid synthase, putative	6.5	3.6	up	0.007
EHI_059320_s_at	XM_001914076	hypothetical protein	2.6	3.4	up	0.037
EHI_105080_at	XM_648821	zinc finger protein, putative	8.2	3.4	up	0.026
EHI_126560_at	XM_001914189	AIG1 family protein, putative	7.5	3.4	up	0.004
EHI_091670_at	XM_644055	deoxyuridine 5′triphosphate nucleotidohydrolase domain containing protein	2.3	3.4	up	0.030
EHI_180390_at	XM_648725	AIG1 family protein, putative	9.1	3.4	up	0.016
EHI_139400_at	XM_646219	TATA-binding protein-associated phosphoprotein, putative	3.7	3.3	up	0.002
EHI_154270_at	XM_645351	cell division control protein 42, putative	6.1	3.3	up	0.008
EHI_172510_at	XM_643770	acid sphingomyelinase-like phosphodiesterase 3a precursor, putative	3.5	3.2	up	0.000
EHI_050570_at	XM_651510	cysteine proteinase, putative	11.3	3.2	up	0.001
EHI_052130_at	XM_650257	PQ loop repeat protein	4.6	3.1	up	0.016
EHI_009910_at	XM_652020	TBC domain containing protein	5.6	3.0	up	0.011
EHI_121750_at	XM_646688	hypothetical protein	5.5	3.0	up	0.006

**Table 3:** List of genes down and up regulated ≥3 fold upon SAT3 gene silencing.

ProbeSetID	Accession Number	Common Name	Basal Expres-sion (log_2_)	Fold change	Regula-tion	p-value
EHI_153430_at	XM_651281	serine acetyltransferase 3	10.5	186.7	down	0.007
EHI_153420_at	XM_651282	hypothetical protein	7.9	14.0	down	0.011
EHI_196760_s_at	XM_643708	hypothetical protein	7.7	12.5	down	0.004
EHI_020830_s_at	XM_001913952	hypothetical protein	7.8	7.6	down	0.002
EHI_128180_s_at	XM_649666	Rab family GTPase	8.1	6.5	down	0.004
EHI_147860_at	XM_646798	hypothetical protein	5.6	5.2	down	0.011
EHI_133210_s_at	XM_001914244	peptidase S54 (rhomboid) family protein	7.9	5.1	down	0.011
EHI_051430_at	XM_652271	Ras guanine nucleotide exchange factor, putative	5.0	4.6	down	0.032
EHI_128190_s_at	XM_649665	peptidase S54 (rhomboid) family protein	5.8	4.5	down	0.005
EHI_079870_at	XM_647774	NTP pyrophosphatase domain containing protein	7.3	4.1	down	0.024
EHI_164410_at	XM_649301	DNA double-strand break repair Rad50 ATPase, putative	4.4	3.9	down	0.008
EHI_111210_at	XM_652499	DNA double-strand break repair Rad50 ATPase, putative	4.2	3.6	down	0.000
EHI_178130_at	XM_646412	hypothetical protein	8.8	3.5	down	0.002
EHI_079970_at	XM_001913704	leucine rich repeat protein, BspA family	5.2	3.2	down	0.027
EHI_005930_at	XM_648325	hypothetical protein	4.1	3.1	down	0.011
EHI_161970_at	XM_644065	leucyl-tRNA synthetase, putative	8.7	3.1	down	0.010
EHI_170940_at	XM_001913890	lipase, putative	8.9	3.1	down	0.002
EHI_074480_s_at	XM_001914032	hypothetical protein	3.9	3.0	down	0.002
EHI_054660_at	XM_646970	apyrase, putative	3.9	3.0	down	0.047
EHI_022600_s_at	XM_643169	NADPH-dependent FMN reductase domain containing protein	7.0	8.9	up	0.002
EHI_022270_s_at	XM_644774	NADPH-dependent FMN reductase domain containing protein	7.2	8.5	up	0.002
EHI_067720_s_at	XM_643101	NADPH-dependent FMN reductase domain containing protein	7.3	8.3	up	0.001
EHI_181710_s_at	XM_001914510	NADPH-dependent FMN reductase domain containing protein	7.3	7.9	up	0.004
EHI_103260_s_at	XM_001913434	NADPH-dependent FMN reductase domain containing protein	7.3	7.2	up	0.001
EHI_153710_at	XM_001913338	methylene-fatty-acyl-phospholipid synthase, putative	6.5	5.8	up	0.002
EHI_029930_at	XM_001914435	hypothetical protein	3.6	4.7	up	0.003
EHI_155430_s_at	XM_650657	hypothetical protein	4.7	4.0	up	0.028
EHI_091670_at	XM_644055	deoxyuridine 5′triphosphate nucleotidohydrolase domain containing protein	2.3	3.9	up	0.007
EHI_146130_at	XM_644793	hypothetical protein	3.3	3.8	up	0.000
EHI_072960_s_at	XM_001914509	deoxyuridine 5′triphosphate nucleotidohydrolase domain containing protein	4.3	3.7	up	0.011
EHI_158010_at	XM_645706	hypothetical protein	4.0	3.5	up	0.024
EHI_034530_s_at	XM_643791	hypothetical protein	2.5	3.4	up	0.036
EHI_074750_at	XM_644490	Ras family GTPase	5.5	3.4	up	0.011
EHI_198440_s_at	XM_001914343	hypothetical protein	2.5	3.3	up	0.013
EHI_018140_s_at	XM_001914260	deoxyuridine 5′-triphosphate nucleotidohydrolase domain containing protein	5.5	3.3	up	0.016

### Confirmation of differential gene expression by qRT–PCR

The microarray results were validated by qRT–PCR. Table 4 shows a comparison of the qRT-PCR and microarray data of six representative differentially expressed genes identified by the transcriptome analysis, with the RNA polymerase II gene used as reference^33^. The results of the qRT-PCR analysis agreed well with the microarray data for all examined gene transcripts (Table 4).

**Table 4:** Validation of microarray data by qRT-PCR and microarray analysis.

Common Name	Accession Number	Fold Change by qRT-PCR (by microarray)		
		SAT1/2gs	SAT3gs	CSgs
Fe-hydrogenase	XM_647045	5.0 (4.4)	ND	ND
Sulfotransferase	XM_648447	9.1 (8.5)	ND	ND
Phosphoserine aminotransferase	XM_650291	−4.6 (−5.1)	ND	ND
NADPH-dependent oxidoreductases 2	XM_648481	1.4 (1.9)	1.3 (1.7)	4.7 (4.0)
NADPH-dependent FMN reductase domain-containing protein	XM_643169	2.4 (3.0)	9.4 (8.9)	5.1 (4.0)
Methylene-fatty-acyl-phospholipid synthase	XM_001913338	4.1 (3.6)	4.7 (5.8)	4.5 (3.9)
RNA polymerase II	XM_643999	1.2 (1.4)	1.2 (1.1)	1.1 (1.0)

The common names, accession numbers, and fold changes of the selected genes are shown. The values are the fold changes in the expression obtained from qRT-PCR and the corresponding fold changes in the expression values obtained from Affymetrix analysis are shown in parentheses. ND, not detected.

### S-Methylcysteine production leads to increased oxidative stress tolerance

**Figure 4:**
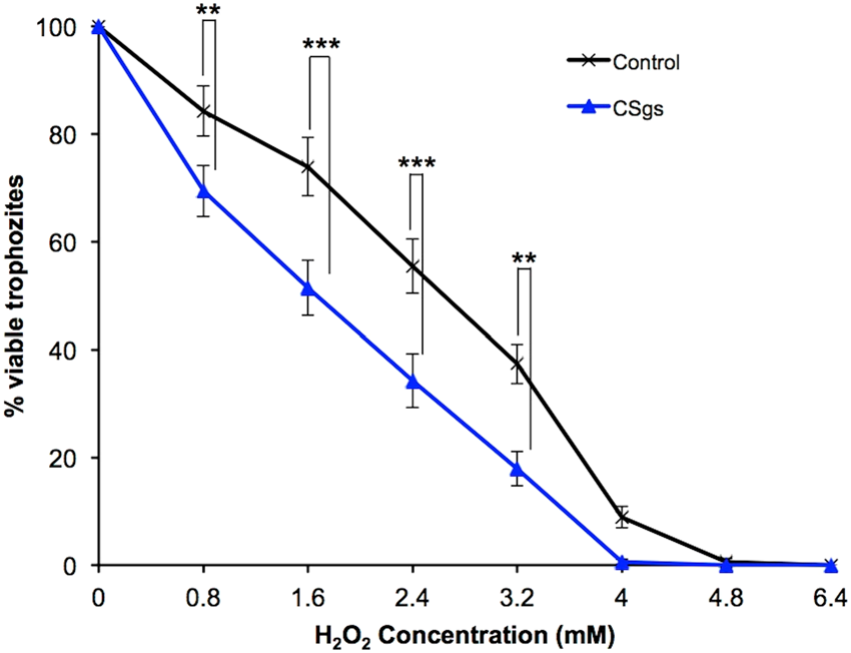
Effect of *CS* gene silencing on oxidative stress tolerance. Trophozoites of CSgs and control (harboring plasmid psAP2G) strains were exposed to different concentrations of H_2_O_2_ for 1 h and viability was then determined. Survival rates are shown as percent of untreated control cells (mean ±S.D. of three independent experiments conducted in triplicate). Statistical comparisons were made by the Student’s t test (**P <0.01, ***P <0.001).

To investigate whether the SMC accumulation observed in trophozoites cultured in L-cysteine lacking BI-S-33 medium protects *E*. *histolytica* against oxidative stress, the CSgs, which does not produce SMC and control (harboring plasmid psAP2G) transformants were compared for oxidative stress sensitivity by culturing the two strains in L-cysteine lacking medium. After 48-h cultivation in L-cysteine lacking medium, SMC had accumulated in the control transformant, but remained absent in the CSgs transformant (Fig. 3). The CSgs and control transformants were next exposed to different concentrations of H_2_O_2_ (0–6.4 mM) for 1 h and viability was then determined. The CSgs transformant showed slightly, but significantly (Student’s t-test), lower sensitivity to 0.8–4.0 mM H_2_O_2_ compared to the control transformant, suggesting that CS1–3 or SMC synthesis may be involved in protecting the cells against oxidative stress (Fig. 4).

## Discussion

The identification and functional characterization of the molecular components involved in essential metabolic pathways contribute to the overall understanding of parasite biology, but also aid in the rational design of novel therapeutics. L-Cysteine is indispensable for the survival of virtually all living organisms and plays a major role in maintaining the redox balance of thiol compounds in microaerophiles^18^. The cysteine biosynthetic pathway exists in bacteria, plants, and several parasitic protozoa, including *Leishmania major*, *Trypanosoma cruzi*, and *Trichomonas vaginalis* ^1^, and enzymes involved in this pathway are suitable targets for the development of novel drugs to prevent disease caused by these parasites^2–6^.

In the present study, we investigated the specific role of individual isotypes of SAT and CS using a gene silencing approach. Although SAT^26^ and CS^35^ have been biochemically^13–16^ and structurally characterized, the specific role of individual SAT and CS isotypes in proliferation, pathogenesis, and parasitism remains to be elucidated. Although we attempted to silence the expression of single genes, all the three CS isotypes were simultaneously silenced in *E*. *histolytica* due to their high similarity at the nucleotide and amino acid levels (CS1-3, 83–99%). The global repression of CS expression resulted in impaired trophozoites proliferation in L-cysteine lacking BI-S-33 medium, but not in normal BI-S-33 medium containing 8 mM L-cysteine (Fig. 2B). Metabolomic analysis of the *CS*-silenced transformant under the two culture conditions further revealed that SMC was not detectable and that the level of OAS was markedly reduced, demonstrating that CS is involved in SMC synthesis from OAS (Fig. 3). We previously showed that *E*. *histolytica* trophozoites produce SMC, rather than L-cysteine, when cultured in L-cysteine lacking BI-S-33 medium^13^. The present metabolomic analysis further revealed that L-cysteine levels were also decreased when the CSgs (and SATgs) transformants were cultured in normal BI-S-33 medium containing 8 mM L-cysteine, suggesting that *E*. *histolytica* synthesizes both L-cysteine and SMC, and that the flux towards cysteine synthesis likely depends upon the intracellular availability of sulfides (i.e., methanethiol and sulfide).

In contrast to *CS*, silencing of *SAT1/2* and *SAT3* was specific to the targeted SAT isotypes. Unlike other organisms, *E*. *histolytica* possesses three apparently redundant SAT isozymes^16^. These three SAT isotypes differ from one other in their regulatory properties. The isoenzymes SAT1 and SAT2 are regulated through allosteric feedback by L-cysteine^15,16^, whereas SAT3 is relatively insensitive to L-cysteine inhibition^16^. Consistent with these findings, EhSAT1-3 showed different levels of sensitivity to allosteric feedback by L-cysteine^16^ [inhibition constant (Ki) values of EhSAT1-3 are 4.7, 28, and 460 μM, respectively]. We previously showed that under cysteine lacking conditions, OAS and SMC expression levels in *E*. *histolytica* increase, whereas the expression of SAT and CS isotypes are not affected by L-cysteine depletion^13,36^. As OAS and SMC were undetectable under normal conditions, OAS, which is produced by SAT1-3, appears to be readily converted to cysteine, but not SMC. Alternatively, the *in-vivo* activities of SAT3 and cysteine-sensitive SAT1/2 may be repressed by unknown mechanisms. Under cysteine lacking conditions, L-cysteine-sensitive SAT1 and SAT2, together with cysteine-insensitive SAT3, were derepressed, leading to increased production of OAS. The mechanism by which SMC, but not cysteine, accumulates in response to cysteine deprivation in strains SAT1/2gs and SAT3gs remains unknown. However, it is conceivable that in strain SAT1/2gs, SAT3 compensates for the loss of SAT1/2 by producing sufficient cellular OAS and thereby contributes to the maintenance of high SMC levels under cysteine lacking conditions.

Another unique aspect of *E*. *histolytica* SAT1 is the lack of protein-protein interaction with CS^26^. It is well known that in bacteria and plants, CS and SAT form a heteromeric complex with a molecular mass of several hundred kilodaltons^37^. However, EhCS1 and EhSAT1 form a homodimer and homotrimer, respectively^26^, but these proteins do not interact under physiological conditions^26^. The lack of interaction between EhCS1 and EhSAT1 was structurally elucidated^26,35^. The apparent reduction of cysteine/SMC production in SAT3gs strain despite high level of OAS may explain the possible formation of a SAT3-CS complex that regulates cellular CS activity. Here, silencing of SAT3 resulted in the loss of complex formation, reduction of CS activity, and decreased production of SMC and L-cysteine (Fig. 3). Metabolomic analysis confirmed that the similar response occurred in the *CS* gene-silenced transformants. SAT3 possesses a unique 25–30 amino acids extension at the carboxyl terminus and has a low isoelectric point compared to SAT1 and SAT2^16^. These features may favor the interaction with *E*. *histolytica* CS, particularly EhCS3, which possesses the highest pI (8.17) among the three CS isotypes. However, this hypothesis needs to be experimentally proven.

The present metabolomic analyses combined with the results of the growth kinetic assay demonstrated that neither the concentrations of OAS, L-cysteine or SMC in the two culture conditions, nor trophozoites growth under L-cysteine lacking conditions were affected by SAT1/2 gene silencing (Fig. 3). These data suggest that SAT3 is a robust enzyme that likely compensated for the loss of SAT1/2 under *in-vitro* conditions. In contrast to SAT1/2, repression of SAT3 had more marked effects on growth than the repression of SAT1/2, suggesting that SAT3 is critical for survival under stressful conditions, whereas SAT1/2 are involved in more general house-keeping roles. This speculation is also supported by the fact that the levels of both L-cysteine and SMC were decreased in strain SAT3gs.

To determine whether other genes, particularly those involved in sulfur metabolism, compensate for the loss of CS and SAT gene expression, we compared the transcriptomes of the CSgs, SAT1/2, SAT3gs, and control strains grown in normal medium. Notably, several genes from a family of the NADPH-dependent FMN reductase domain-containing proteins, also known as iron-sulfur flavoproteins (ISFs), which are commonly found in anaerobic prokaryotes, were highly upregulated in CSgs and SAT3gs (Tables 1 and 3). To date, the only eukaryotic species that have been found to possess ISF homologs are *E*. *histolytica* and *Trichomonas vaginalis* ^38,39^. A search of the genome database of *E*. *histolytica* revealed the presence of seven independent ISF genes^40^, which were previously shown to be upregulated in *E*. *histolytica* cells cultured in L-cysteine lacking BI-S-33 media, suggesting that these genes are regulated in response to L-cysteine deprivation^36^.

In contrast to CSgs strain, we found that in SAT1/2 gene-silenced strain one of the sulfotransferase, *SULT9* (XP_653539, EHI_031640) (Table 2), was up-regulated more than eight fold, suggesting its involvement in L-cysteine biosynthesis and/or redox-related metabolism. The *E*. *histolytica* genome contains 10 genes that encode putative sulfotransferases (SULTs), which are localized in the cytosol and are involved in the production of sulfated molecules^41^. For example, *SULT6* (XP_649714, EHI_146990) is responsible for synthesizing cholesteryl sulfate, an important compound for the encystation process in the *Entamoeba* life cycle^41^. However, the function of other SULTs in *E*. *histolytica* remains largely unknown. In *Arabidopsis* roots, a plasma membrane sulfate ion transporter (SULTR) physically interacts with CS to coordinate internalization of sulfate ions based on the energetic/metabolic state of root cells^42^. Here, we also determined that Fe hydrogenase, which belongs to a distinct class of hydrogen-producing metalloenzymes and is found in a wide variety of prokaryotes and eukaryotes^43^, was up-regulated more than four fold in strain SAT1/2gs. Fe hydrogenase contributes to the utilization of hydrogen as a growth substrate and for the disposal of excess electrons through combination with protons to form hydrogen^43^. Although the role of Fe hydrogenase in *Entamoeba* is unclear, it is possible that this enzyme is regulated in response to oxygen levels, as was shown in *Chlamydomonas reinhardtii*, which contained increased transcript levels of Fe hydrogenase upon shifting from an aerobic to anaerobic atmosphere^44^.

The present metabolome data of strain CSgs suggest that in addition to L-cysteine, CS enzymes are involved in SMC production (Fig. 3). SMC is a sulfur-containing amino acid that is found in relatively large amounts in several legumes, where it is considered to be a sulfur storage compound^45^. However, the fate and physiological significance of SMC in protozoa, particularly *E*. *histolytica*, is not yet fully understood. Previously, we investigated metabolic responses to hydrogen peroxide - and paraquat-mediated oxidative stress in *E*. *histolytica* trophozoites and reported that SMC levels are increased more than two fold under both stress conditions^46^, suggesting the involvement of this metabolite in the oxidative stress response. To confirm this speculation, we compared the oxidative stress tolerance between the control and CSgs transformant because SMC was undetected in CSgs strain (Fig. 3), and demonstrated that the CSgs transformant was more sensitive to oxidative stress. In *Brassica* exposed to H_2_O_2_ or O_2_ stress, SMC is non-enzymatically converted to SMC sulfoxide^47^, which is further enzymatically catabolized into pyruvate, ammonia, and alkylthiosulfinates^48^. The enzyme that catalyzes the last reaction is cystine lyase (EC 4.4.1.8) and behaves similarly to allinase (EC 4.4.1.4) in garlic, with the exception that cystine lyase also has the ability to cleave L-cystine^49^. Based on this observation, we propose that under oxidative stress conditions, SMC is converted to SMC sulfoxide and is further degraded by a lyase enzyme, such as methionine γ-lyase^17^ (MGL), to pyruvate and sulfenic acid.

In summary, the present metabolomic analysis revealed that CS and SAT3 are key enzymes for cysteine/SMC production in *E*. *histolytica* and are also essential for parasite survival under oxidative stress conditions. Transcriptomic analysis of the constructed CSgs and SAT3gs strains revealed that compensatory mechanisms in which ISFs play key roles operate under conditions where the CS and SAT3 pathway(s) are inactivated. These findings corroborate the metabolic and physiological importance of the L-cysteine pathway in *E*. *histolytica* and suggest that CS and SAT3 represent good targets for drug development. Further work is needed to demonstrate the specific role of these ISFs in *E*. *histolytica*.

## Methods

### Microorganisms and cultivation

*In-vitro* cultures of *E*. *histolytica* strains HM-1:IMSS cl6 and G3 were routinely maintained in Diamond’s BI-S-33 medium at 35.5 °C, as described previously^50,51^.

### Gene silencing

Strain G3 and plasmid psAP2 were kindly provided by Dr. David Mirelman (Weisman Institute, Israel)^28,29^. Gene silencing was performed as previously described^28,29,36,52^. Briefly, 420-bp fragments containing the entire open reading frames of the *E*. *histolytica CS1*, *CS3*, *SAT1 SAT2* and *SAT3* genes starting at the initiation codon were amplified by PCR from cDNA using the oligonucleotide primers listed in Supplementary Table S1. The obtained PCR products were digested with StuI and SacI and inserted into StuI/SacI-digested pSAP2G to produce psAP2G-CS1, psAP2G-CS3, psAP2G-SAT1, psAP2G-SAT2 and psAP2G-SAT3. The constructed plasmids were introduced into *E*. *histolytica* strain G3 by liposome-mediated transfection^15^, and the resulting transformants (designated psAP2G [control], CS1gs, CS3gs, SAT1gs, SAT2gs and SAT3gs) were selected and maintained in normal BI-S-33 medium supplemented with 7 μg/ml geneticin (Invitrogen). The expression of the respective genes was confirmed by semi-quantitative RT-PCR as described previously using RNA polymerase II mRNA (GenBankTM accession number XM_643999) as a reference^33^, as the expression of this gene was invariant in all of the transformants. The transformants were designated psAP2G (control), CS1gs, CS3gs, SAT1gs, SAT2gs and SAT3gs.

### Extraction of metabolites from *E*. *histolytica*

*E*. *histolytica* trophozoites were cultured for approximately 24 h in standard BI-S-33 medium containing 8 mM L-cysteine. The medium was replaced with either normal BI-S-33 medium or medium lacking L-cysteine^13^, and trophozoites were cultured for a further 48 h. To extract metabolites, approximately 1.5 × 10^6^ cells were then harvested and immediately suspended in 1.6 mL of −75 °C methanol to quench metabolic activity. To minimize the effects of experimental artifacts, such as ion suppression, on metabolite levels, 2-(N-morpholino) ethanesulfonic acid, methionine sulfone, and D-camphor-10-sulfonic acid were added to each sample as internal standards^13,53,54^. The samples were sonicated for 30 s and then mixed with 1.6 mL chloroform and 640 µl deionized water. After vortexing, the mixed samples were centrifuged at 4600 g for 5 min at 4 °C. The aqueous layer (1.6 mL) was filtrated using an Amicon Ultrafree-MC ultrafilter (Millipore Co., Massachusetts, USA) and the collected sample was centrifuged at 9100 g at 4 °C for approximately 2 h. The filtrate was vacuum dried and stored at −80 °C until needed for mass spectrometric analysis^55^. Prior to the analysis, the sample was dissolved in 20 μl de-ionized water containing 200 μmol/L of two reference compounds (3-aminopyrrolidine and trimesic acid).

### Instrumentation and capillary electrophoresis-time-of-flight mass spectrometry (CE-TOFMS)

CE-TOFMS was performed using an Agilent CE Capillary Electrophoresis System equipped with an Agilent 6210 Time-of-Flight mass spectrometer, Agilent 1100 isocratic HPLC pump, Agilent G1603A CE-MS adapter kit, and Agilent G1607A CE-ESI-MS sprayer kit (Agilent Technologies, Waldbronn, Germany). The system was controlled by Agilent G2201AA ChemStation software for CE. Data acquisition was performed using Analyst QS software for Agilent TOF (Applied Biosystems, CA, USA; MDS Sciex, Ontario, Canada).

### CE-TOFMS conditions for cationic metabolite analysis

Cationic metabolites were separated in a fused-silica capillary (50 μm i.d. × 100 cm total length) filled with 1 mol/L formic acid as the reference electrolyte^56^. Sample solution (~3 nL) was injected at 50 mbar for 3 s, and a positive voltage of 30 kV was applied. The capillary and sample trays were maintained at 20 °C and below 5 °C, respectively. Sheath liquid composed of methanol/water (50% v/v) and 0.1 μmol/L hexakis (2,2- difluorothoxy) phosphazene was delivered at 10 μL/min. ESI-TOFMS was operated in positive ion mode. The capillary voltage was set at 4 kV and the flow rate of nitrogen gas (heater temperature 300 °C) was set at 10 psig. For TOFMS, the fragmenter voltage, skimmer voltage, and octapole radio frequency voltage (Oct RFV) were set at 75, 50, and 125 V, respectively. An automatic recalibration function was performed using the masses of two reference compounds, protonated 13C methanol dimer (m/z 66.063061) and protonated hexakis (2,2-difluorothoxy) phosphazene (m/z 622.028963), which provided the lock mass for exact mass measurements. Exact mass data were acquired at the rate of 1.5 cycles/s over 50 to 1,000 m/z.

### CE-TOFMS conditions for anionic metabolite analysis

Anionic metabolites were separated in a cationic-polymer–coated COSMO(+) capillary (50 μm i.d. × 110 cm) (Nacalai Tesque) filled with a 50 mmol/L ammonium acetate solution (pH 8.5) as the reference electrolyte^57,58^. Sample solution (~30 nL) was injected into the system at 50 mbar for 30 s and a negative voltage of −30 kV was applied. Ammonium acetate (5 mmol/L) in methanol/water (50% v/v) containing 0.1 μmol/L hexakis (2,2-difluorothoxy) phosphazene was delivered as sheath liquid at 10 μL/min. ESI-TOFMS was performed in negative ion mode at a capillary voltage of 3.5 kV. For TOFMS, the fragmenter voltage, skimmer voltage, and Oct RFV were set at 100, 50, and 200 V, respectively^58^. An automatic recalibration function was performed using the masses of two reference compounds: deprotonated 13C acetate dimer (m/z 120.038339) and an acetate adduct of hexakis (2,2-difluorothoxy) phosphazene (m/z 680.035541). The other conditions were identical to those used for the cationic metabolome analysis.

### CE-TOFMS data processing

Raw data were processed using in-house Masterhands software^59^. The overall data processing flow consisted of the following steps: noise-filtering, baseline-removal, migration time correction, peak detection, and peak area integration from a 0.02 m/z-wide slice of the electropherograms. The data processing resembled the common strategies used for LC-MS and GC-MS data analysis software, such as MassHunter (Agilent Technologies) and XCMS^60^. Accurate m/z values for each peak were calculated by Gaussian curve fitting in the m/z domain, and migration times were normalized using alignment algorithms based on dynamic programming^61,62^. All target metabolites were identified by matching their m/z values and normalized migration times with those of standard compounds in the in-house library.

### RNA isolation and Affymetrix microarray hybridization

Trophozoites were grown in BI-S-33 medium containing 8 mM L-cysteine for approximately 48 h. The collected cell pellets were resuspended in Trizol reagent (Invitrogen, Carlsbad, CA, USA) and RNA was isolated according to the manufacturer’s protocol. The RNA concentration for each sample was measured using a Nanodrop Spectrophotometer 1000 (Thermo Scientific, Wilmington, DE, USA). RNA integrity was checked using an Experion Automated Electrophoresis System (RNA StdSens analysis kit, Bio-Rad). All reagents and protocols followed those described in the Affymetrix user manuals. Using the One-Cycle cDNA synthesis kit, 5 μg total RNA was reverse transcribed using a T7-Oligo (dT) primer for first strand cDNA synthesis. After second strand synthesis, the double-stranded cDNA template was used for *in-vitro* transcription (IVT) in the presence of biotinylated nucleotides (GeneChip IVT labeling kit) to produce Biotin-labeled cRNA. Unincorporated NTPs were removed from the biotinylated cRNA (GeneChip sample cleanup module), which was then purified, quantified and fragmented. A hybridization cocktail consisting of eukaryotic hybridization controls and fragmented, labeled cRNA (GeneChip Hybridization, Wash and Stain Kit) were hybridized for 16 h at 45 °C in a Hybridization Oven 640 (Affymetrix) onto a custom-generated Affymetrix platform microarray (49–7875) with probe sets consisting of 11 probe pairs, each representing 12,384 *E*. *invadens* ^63^ (Eh_Eia520620F_Ei) and 9,327 *E*. *histolytica* ^36^ (Eh_Eia520620F_Eh) open reading frames. The array chips were washed and stained (GeneChip Hybridization, Wash and Stain Kit) with Streptavidin–phycoerythrin Biotinylated anti-streptavidin antibody using a GeneChip Fluidics Station 450 (Affymetrix) for 1.5 h. After washing and staining, the GeneChip arrays were scanned using a Hewlett-Packard Affymetrix Scanner 3000.

### Analysis of microarray data

A minimum of two arrays was used for each test condition. Raw probe intensities were generated using Gene Chip Operating Software (GCOS) and the Gene Titan Instrument from Affymetrix. Normalized expression values for each probe set were obtained from R 2.7.0 downloaded from the Bioconductor project (http://www.bioconductor.org) using robust multiarray averaging with correction for oligosequence (gcRMA). Standard correlation coefficients were calculated using GeneSpring GX 10.0.2. One-way ANOVA analysis with Tukey’s Post Hoc test was performed to extract differentially expressed genes. P values were calculated using Welch’s t-test after multiple test correction by the Benjamini–Hochberg method. A post-hoc test using Tukey’s Honestly Significant Difference test was conducted to determine significant differences between samples.

### Quantitative real-time PCR (qRT–PCR)

Total RNA extracted above were used for qRT–PCR. cDNA synthesis was performed using the SuperScript III First-Strand Synthesis System (Invitrogen) following the manufacturer’s instructions. cDNA was synthesized from 5 μg total RNA and oligo (dT) 20 primers using the Superscript III First-Strand Synthesis System (Invitrogen). PCR was performed with cDNA as the template and gene-specific primers using the ABI PRISM 7300 Sequence Detection System (Applied Biosystems, Weiterstadt, Germany). The genes whose expression was verified by qRT–PCR are listed in Supplementary Table S4. The RNA polymerase II gene was used as a control. The parameters for PCR were: an initial denaturation step at 95 °C for 9 min followed by 40 cycles of denaturation at 94 °C for 30 s, annealing at 50 °C for 30 s and extension at 65 °C for 1 min. A final step at 95 °C for 9 s, 60 °C for 9 s and 95 °C for 9 s was used to remove primer dimers. All test samples were run in triplicate. An RT-negative control was also used for each sample set along with a blank control consisting of nuclease-free water in place of cDNA.

### Growth assay of *E*. *histolytica* trophozoites

A cell-growth assay was performed as described previously^36^. Briefly, approximately 6 × 10^4^ exponentially growing SAT1/2, SAT3, or CS gene-silenced trophozoites and control transformants were inoculated into 6 mL normal BI-S-33 medium with and without L-cysteine supplemented with 7 μg/mL geneticin, and the number of parasites was counted every 24 h using a haemocytometer.

### Hydrogen peroxide (H_2_O_2_) sensitivity assay

To examine sensitivity to H_2_O_2_, *E*. *histolytica* CS gene-silenced and control (harboring plasmid psAP2G) transformants were cultured in L-cysteine lacking BI-S-33 media containing 7 μg/mL geneticin for 48 h at 35.5 °C. After 48 h, approximately 10^4^ trophozoites per well were seeded into the wells of a 96-well plate containing BI-S-33 medium supplemented with 7 μg/mL geneticin and further incubated for 1 h at 35.5 °C. The trophozoites were then exposed to H_2_O_2_ (0, 0.8, 1.6, 2.4, 3.2, 4, 4.8 and 6.4 mM) for 1 h. After incubation, the medium was removed and 90 μl of pre-warmed Opti-MEM I (Life Technologies) and 10 μl WST-1 solution^64^ (Roche Diagnostics, Mannheim, Germany) were added to each well. Viability of trophozoites was detected by measuring absorbance at 450 nm using a microplate reader (Model 550, Bio-Rad, Tokyo, Japan). The sensitivity assays were performed in triplicate and repeated at least three times.

## Electronic supplementary material

Supplementary Fig. S1

Dataset 1

Dataset 2

Dataset 3

Dataset 4
