# Supplementary material for: Genetic, metabolomic and transcriptomic analyses of the de novo L-cysteine biosynthetic pathway in the enteric protozoan parasite Entamoeba histolytica
Source: Sci Rep. 2017 Nov 15;7:15649. doi: 10.1038/s41598-017-15923-3 (PMC5688106; doi:10.1038/s41598-017-15923-3)

Supplementary Information

Genetic, metabolomic and transcriptomic analyses of the de novo L-cysteine biosynthetic pathway in the enteric protozoan parasite *Entamoeba histolytica*

Ghulam Jeelani[1,2], Dan Sato[3*], Tomoyoshi Soga[3], Tomoyoshi Nozaki[1,2,4]

[1]Department of Biomedical Chemistry, Graduate School of Medicine, The University of Tokyo, 7-3-1 Hongo, Bunkyo-ku, Tokyo 113-0033, Japan

[2]Department of Parasitology, National Institute of Infectious Diseases, 1-23-1 Toyama, Shinjuku, Tokyo 162-8640, Japan

[3]Institute for Advanced Biosciences, Keio University, Tsuruoka, Yamagata, Japan.

[4]Graduate School of Life and Environmental Sciences, University of Tsukuba, 1-1-1 Tennodai, Tsukuba, Ibaraki 305-8572, Japan.

[*]Present address: Graduate School of Science and Technology, Department of Applied Biology, Kyoto Institute of Technology, Kyoto 606-8585, Japan.

Supplementary Fig. S1

Expression of CS proteins in the *SAT1/2 and SAT3* gene-silenced and control strains. Approximately 40 µg of total lysates from the SAT1/2gs, SAT3gs, and control strains (psAP2G) was electrophoresed on an SDS-PAGE gel under reducing conditions and subjected to immunoblot analysis using anti-CS1, anti-CS3, and anti-EhCPBF1 antibodies (left panel). The densitometric quantification of the reacted bands was performed using Image J software. The levels of EhCS1, EhCS3 and EhCPBF1 proteins are expressed in arbitrary units (right panel).

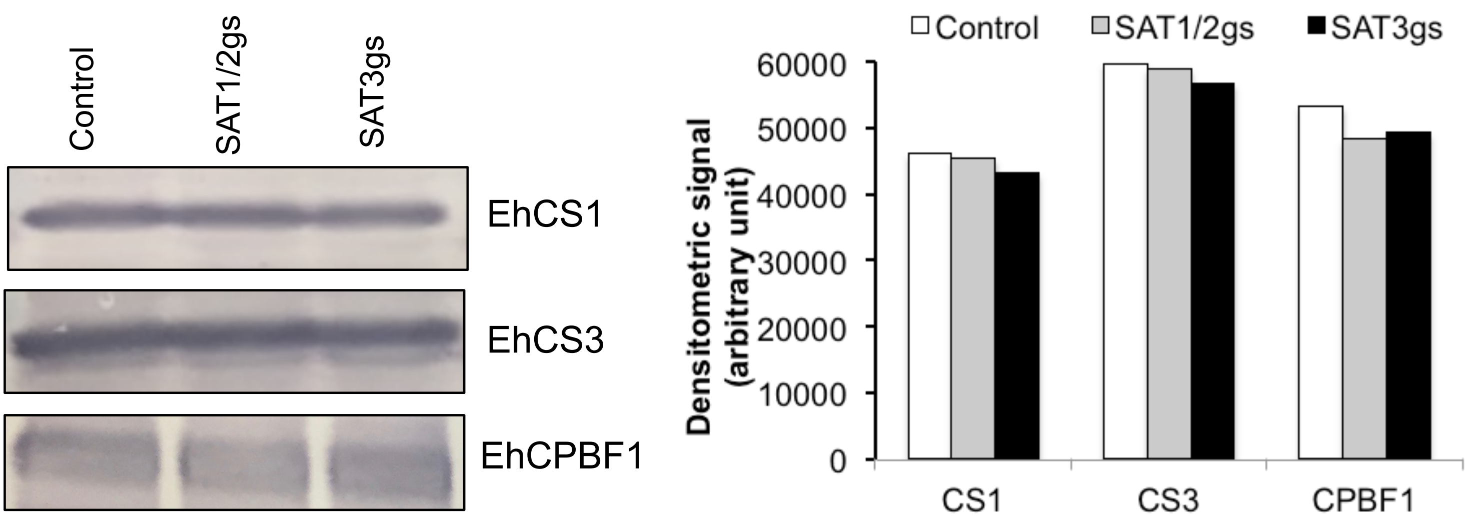

Supplement: Supplementary file 1 — Supplementary Fig. S1 [file 41598_2017_15923_MOESM1_ESM.pdf]
